# Comprehensive Analysis of Improvements in Health-Related Quality of Life and Establishment of QALY Gains in a Government-Funded Bariatric Surgical Program with 5-Year Follow-up

**DOI:** 10.1007/s11695-022-06216-4

**Published:** 2022-07-27

**Authors:** Chiara Chadwick, Paul R. Burton, Jennifer Reilly, Julie Playfair, Cheryl Laurie, Kalai Shaw, Wendy A. Brown

**Affiliations:** 1 Department of Surgery, Central Clinical School, Monash University, Alfred Health, Melbourne, VIC Australia; 2 Oesophago-Gastric and Bariatric Unit, Alfred Health. Melbourne, Victoria, Australia; 3 Department of Anaesthesiology and Perioperative Medicine, Alfred Health, Melbourne, VIC Australia

**Keywords:** Bariatric surgery, HRQoL, QALY, Quality of life

## Abstract

**Purpose:**

Bariatric surgery is an efficacious intervention for substantial and sustained weight reduction in individuals with morbid obesity resulting in health improvements. However, the changes to a patient’s health related quality of life (HRQoL) in the medium to longer term after bariatric surgery have not been adequately characterized. Our aim was to evaluate the change to patient HRQoL 5 years following bariatric surgery in an Australian government-funded hospital system and determine the significance of relationships between change in physical and mental assessment scores and HRQoL utility scores.

**Materials and Methods:**

We performed a longitudinal panel study of 81 adult patients who underwent primary bariatric surgery at an Australian tertiary government-funded hospital and completed multi-attribute utility (MAU), multi-attribute non-utility (MA), and disease-specific adjusted quality of life (AQoL) questionnaires before and after bariatric surgery.

**Results:**

At a mean (SD) 5.72 (1.07) years postbariatric surgery, participants demonstrated statistically significant improvements in mean AQoL-8D utility (0.135 (0.21); *P* < 0.0001), yielding a mean 3.2 (1.67) QALYs gained. Beck Depression Inventory-II scores improved (baseline mean 17.35 (9.57); 5-year mean 14.7 (11.57); *P* = 0.037). Short Form-36 scores improved in the domains of physical functioning and role limitations due to physical health and general health. Change in depression scores and patient satisfaction with surgery were found to be significant predictors of follow up AQoL utility scores.

**Conclusions:**

Bariatric surgery improves physical and psychological quality of life measures over 5 years. The improvement of patient QALYs provide insight to the potential cost utility of publicly funded bariatric surgery in the medium term.

**Graphical abstract:**

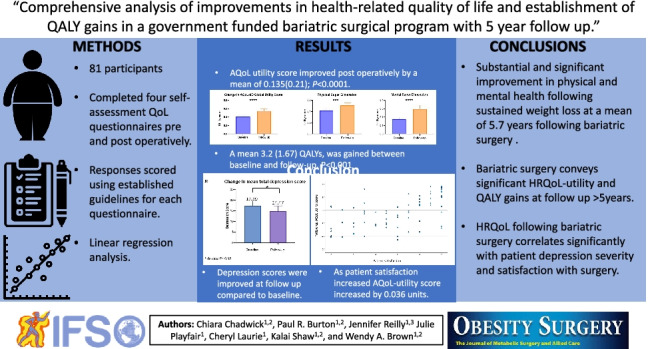

## Background

A primary reason people with obesity seek weight loss through bariatric surgery is to improve quality of life [1]. The adverse effect of obesity and its associated comorbidities on an individual’s quality of life impacts not only physical functioning (exercise tolerance, mobility, musculoskeletal pain) but also has a significant negative impact on their psychosocial wellbeing (reduced social acceptance, clinical mood disorders, reduced sense of self-worth) [2]. Current standard for the management of obesity is multidisciplinary with focus on improving physical and psychosocial wellbeing [3]. Therefore, just as it is important to determine the health benefits of bariatric surgery through remittance of obesity related diseases such as type 2 diabetes mellitus, cardiac dysfunction and reduced fertility, it is equally important to objectively assess the change in quality of life (QoL) as patient-centered outcome measure [4].

Measuring change in QoL following bariatric procedures can facilitate determination of procedural cost effectiveness, which is measured by considering changes in both direct and indirect costs of a disease. Direct costs are those related to treatment of the disease. Indirect costs relate to the loss of productivity as a result of morbidity or mortality from disease, injury, or disability [5, 6]. Direct costs are assessed by summing the cost of the intervention and the changed cost of treatment of the index disease. Indirect costs are assessed by measuring change in health-related quality of life (HRQoL) using quality of life multi-attribute utility (MAU) assessments [7]. Scores derived from MAU assessments can be converted to measures of cost utility represented by quality adjusted life years (QALYs) gained or lost by the population receiving the intervention[11, 12].

MAU assessments are questionnaires that quantify the relative worth of a health intervention as well as the value an individual attributes to the health improvement they receive from the intervention (strength of preference) [8–10]. The scores derived from MAU assessments can be converted to measures of the cost utility of a therapy represented by QALYs gained or lost by the population receiving the intervention [11, 12]. One example of an MAU that is sensitive to changes in QoL following bariatric surgery is the Assessment of Quality of Life (AQoL)-8D [12–14].

Multi-attribute non-utility (MA) assessment tools are descriptive measures of the adverse or harmful effects associated with physical, social, and mental health problems over a period of time. Unlike MAU, MA assessments do not provide a quantifiable measure of the economic impact of a disease state or an intervention [8]. An example of an MA commonly used to describe changes after bariatric surgery is the Medical Outcome Study Short Form–36 (SF-36).

It is well documented that physical health domains of QoL improve after bariatric surgery in the short to mid-term (< 5 years) [15]. However, data on the effect of bariatric surgery on social and psychological health in the short to mid-term are limited and there is little data on any QoL scale beyond 5 years. Publications reporting QoL outcomes 5 years or more after bariatric surgery have to date employed MA quality of life assessment tools [16–18], and therefore cannot give a sensitive estimate of the cost utility of bariatric surgery [13]. There are currently no publications documenting MAU 5 years or more following bariatric surgery.

The total healthcare cost of obesity in Australia was reported in 2017 as AUD $11.8 billion per annum, including AUD $5.4billion in direct and $4.8 billion in indirect costs [6]. Bariatric surgery has previously been shown to be a cost-effective intervention in the short to medium term using data from randomized controlled trials [19]. However, there is currently no data documenting longer term cost utility, nor is there any real-world health-service level data on HRQoL outcomes in recipients of bariatric surgery.

We aimed to evaluate the longer term (> 5 years) impact of bariatric surgery on the QoL of individuals with obesity who had bariatric surgery performed in a public (government funded) hospital setting, and the cost utility of the surgery. We hypothesized that HRQoL would improve 5 years or more after bariatric surgery and this would translate to improved cost utility.

## Method

This was a prospective longitudinal panel study of adult patients who underwent primary bariatric surgery between February 2013 and September 2016. All participants underwent primary bariatric surgery at The Alfred hospital, Melbourne, Australia, one of four state-wide referral centers for public (government-funded) bariatric surgery. All surgery was performed via laparoscopic approach by senior bariatric surgeons by methods previously described [20]. Patient follow-up occurred at The Alfred hospital outpatient bariatric specialist clinic. Ethics approval was obtained from The Alfred hospital Human Research Ethics Committee (Ref 394/12).

### Participant Selection

Participants had completed four established self-assessment QoL questionnaires preoperatively. These included the Assessment of Quality of Life-8 Domains (AQoL-8D), Short form 36 (SF-36), Beck Depression Inventory-II (BDI-II) and the Dakkak score for dysphagia and regurgitation. Consent was sought to retrospectively access these preoperative QoL questionnaire results, and during the consent process, the patients were invited to repeat the questionnaires. Consent was sought over four months from November 2019 to February 2020, and follow-up questionnaires were completed between December 2019 and June 2020.

Eligible individuals were contacted by the investigating team, and informed, voluntary consent for follow-up participation was sought. On initial interview, the patient was informed of the study details, and if they were agreeable, the consent and information forms was sent for perusal. Patients were provided investigator contact details should they have any questions or concerns regarding the study. Participation in the study was only confirmed once the patient had returned the signed copy of their consent form. On receipt of the signed consent form, the participant was sent the questionnaires. If the questionnaires were not returned within one month of initial recruitment a phone call was made to the individual to confirm that they still wished to participate. A second phone call was made 4 weeks later if participants had not returned the questionnaire. Participants were excluded from the study if they failed to return the competed questionnaires 2 months following the second follow-up phone call. If the individual no longer wished to participate, they were removed from the study. At each point of contact, the research team sought to elicit any communication barriers, and if needed, literacy support and interpreter services were offered to the participants.

### Inclusion Criteria

Included patients received their primary bariatric surgery at The Alfred hospital and completed both the baseline and the follow-up questionnaires.

#### Questionnaire Scoring

Scoring of responses was performed using established guidelines for each questionnaire:

AQoL-8D questionnaire responses were converted to utility scores for each domain and an overall AQoL utility score and QALY using the copyrighted AQoL-8D SPSS algorithm published by the Centre for Health Economics Monash University [12]. The minimum important clinical difference (MICD) for the AQoL-8D is 0.03 units [21].

SF-36 questionnaire responses were scored using the RAND corporation scoring system [22]. The MICD for the SF-36 is 5 points [23].

The BDI-II score was calculated as the sum of the response rating for all 21 items. The minimum score is 0 and maximum score is 63. Higher scores indicate greater symptom severity. Scores of 0–13 indicate minimal depression, 14–19 (mild depression), 20–28 (moderate depression), and 29–63 (severe depression) [24]. The MICD for the BDI-II is 17.5% reduction in scores from baseline [25].

The Dakkak score for dysphagia is based on the frequency (never, sometimes, always) of dysphagia experienced when consuming nine different food consistencies, water to meat, scale 0–45. A score < 10 indicates no dysphagia, 10–44 mild dysphagia, and a score > 44 severe dysphagia [26]. There is currently no published MICD for the Dakkak score.

### Patient Satisfaction

Two additional questions were asked regarding overall satisfaction with bariatric surgery and were derived from the validated Short Assessment of Patient Satisfaction (SAPS) questionnaire [27, 28]. These were as follows: “how would you grade your satisfaction with bariatric surgery on a scale of 0–10?” and “how likely would you be to have the bariatric surgery again?”. Satisfaction scores were categorized as dissatisfied (0 to 4), neither satisfied nor dissatisfied (5), and satisfied (6 to 10). Propensity to re-engage in bariatric surgery was measured on an ordinal scale from 1 to 5 (1 = definitely would not, 2 = would most likely not, 3 = unsure, 4 = would most likely again, 5 = would definitely again) and reported as number and percent.

### Statistical Analysis

A sample size of 44 participants was calculated for this two tailed analysis of same subjects, with an effect size of 0.5 and significance of 0.05 to yield a power of 90%. All data were parametric; therefore, Student *t*-test was performed to test our hypothesis. A *P* < 0.05 was considered significant. Pearson’s correlation analysis was performed to determine if there was an association between follow-up AQoL utility score and covariates; total weight loss, change in Dakkak dysphagia score, change in BDI-II score, and patient satisfaction. Linear regression was performed to determine the strength of relationship between follow-up risk covariates and AQoL utility score. All statistical analyses were performed using IBM SPSS version 22 (SPSS Inc, Chicago, IL, USA).

## Results

### Study Population

One hundred eighty adults consented to investigators accessing baseline questionnaire results and to complete follow up questionnaires. Of these, 99 individuals failed to return their postoperative questionnaires 4 months after routine study follow-up and two further contact attempts by the research team. The remaining 81 individuals were included in our final study population.

At baseline participants had a mean (SD) BMI of 48.12 kg/m^2^ (8.45) and a mean age of 47 years (10.04). The study population was predominantly female (75.6%) and 94% of the bariatric procedures performed were laparoscopically inserted adjustable gastric band (LAGB) (Table 1). At baseline the patients had a mean of 11.7 (4.9) comorbidities compared with a mean of 9.0 (4.5) at follow-up, *P* < 0.001 (Table 2).

**Table 1:** Participant demographics

	Number
Gender	
Male	20 (24.7%)
Female	61 (75.3%)
Age at follow-up	
Mean (SD)	52.25 years (10.49)
Follow-up time	
Mean (SD)	5.72 years (1.07)
Baseline weight	
Mean (SD)	130.59 kg (25.69)
Baseline BMI	
Mean (SD)	48.12 kg/m^2^ (8.45)
Follow-up weight	
Mean (SD)	112.7 kg (29.19)
Follow-up BMI	
Mean (SD)	41.88 kg/m^2^ (11.21)
Total body weight loss	
Mean (SD)	18.43 kg (20.61)
%TWL	
Mean (SD)	13.57% (15.02)
Bariatric procedure	
LAGB	76
LSG	4
GB	0
BPD	1

**Table 2:** Participant comorbidities

Comorbidity	Baseline (*n*)	Follow-up (*n*)	*P* (chi-square)
Allergy			
Hayfever	31	30	0.92
Cardiovascular			
Hypertension	47	51	0.45
Dyslipidemia	30	31	0.77
Cardiac failure	5	9	0.25
Angina	6	4	0.54
Endocrine			
Diabetes	30	30	0.9
Polycystic ovarian syndrome	19	14	0.39
Thyroid	8	11	0.41
Gastrointestinal			
Gastroesophageal reflux	42	54	0.06
Gallstones	10	13	0.52
Fatty Liver disease	4	7	0.33
Peptic ulcer disease	4	5	0.70
Genitourinary			
Renal disease	9	10	0.69
Urinary incontinence	13	15	0.62
Musculoskeletal			
Arthritis	37	46	0.23
Back pain	65	57	0.23
Leg pain	59	59	0.89
Other joint pain	2	8	0.06
Neurological			
Recurrent headaches	37	28	0.19
Psychological			
Depression/anxiety	46	42	0.84
Respiratory			
Asthma	41	29	0.06
OSA with CPAP	20	24	0.50
OSA no CPAP	20	23	0.50
Skin integrity			
Skin fold irritation/rashes/ulcers	25	25	1.0
Leg ulcers	32	20	0.06

The mean (SD) duration between surgery and completion of postoperative questionnaires was 5.72 years (1.07). At follow-up, 92.6% of respondents self-reported their current weight. These participants had a lost a mean 18.43 kg (20.61), *P* < 0.0001, and had a mean BMI of 41.88 kg/m^2^ (11.21), representing a mean total percentage weight loss (%TWL) of 13.57% (15.02).

### AQoL-8D

**Fig. 1:**
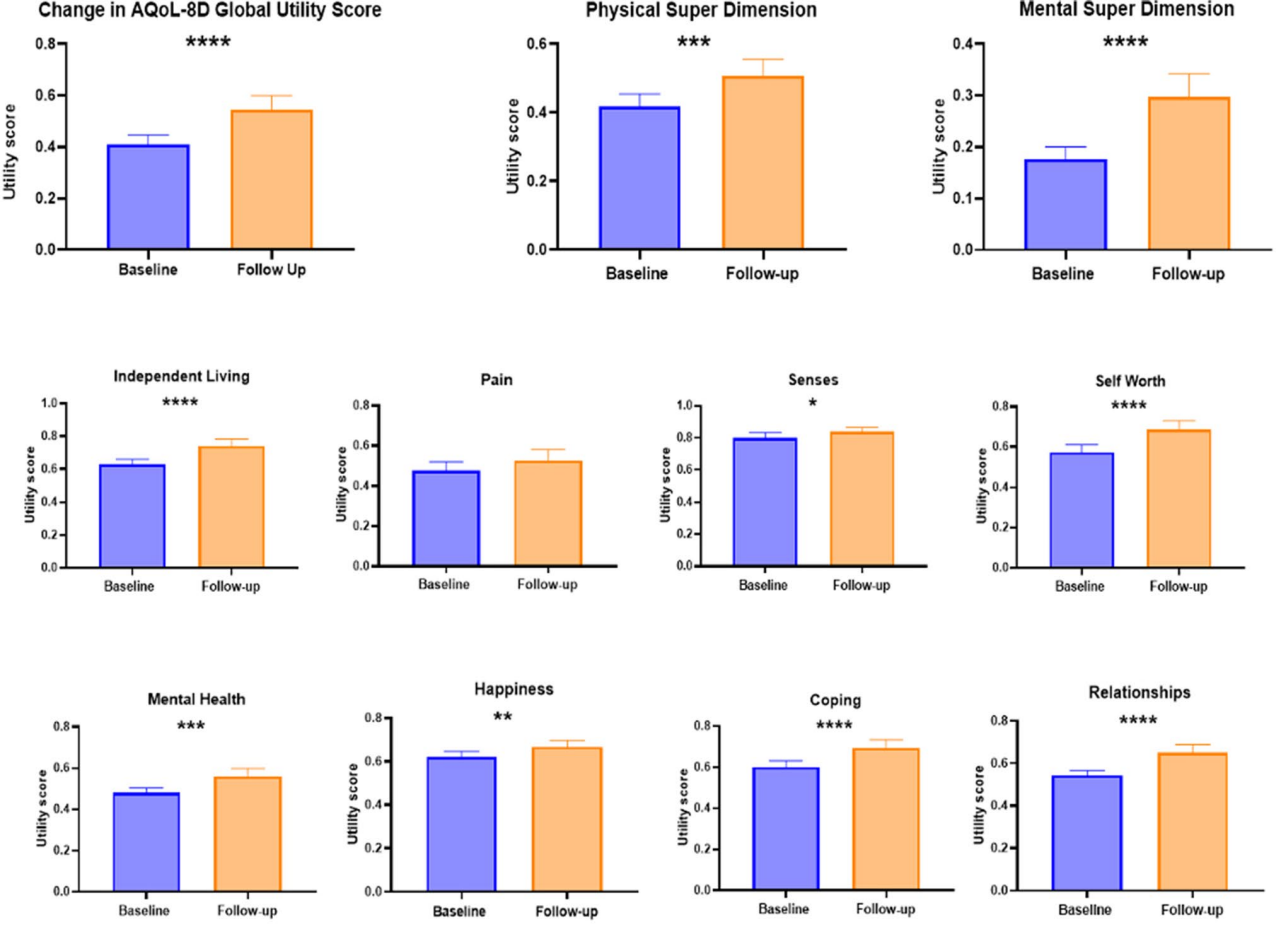
Comparison of baseline and follow-up mean AQoL-8D weighted utility scores. ****P < 0.001, ***P < 0.005, **P < 0.01, *P < 0.05

Statistically significant improvements were observed postoperatively in seven of the eight AQoL dimensions (Fig. 1). The increase in utility score for Dimension 7 “Pain” was not significant (*P* = 0.09). The overall AQoL utility score improved postoperatively by a mean of 0.135 (0.21), *P* < 0.0001. An average 3.2 (1.67) QALYs was gained between baseline and follow-up, *P* < 0.001.

### RAND SF-36

**Fig. 2:**
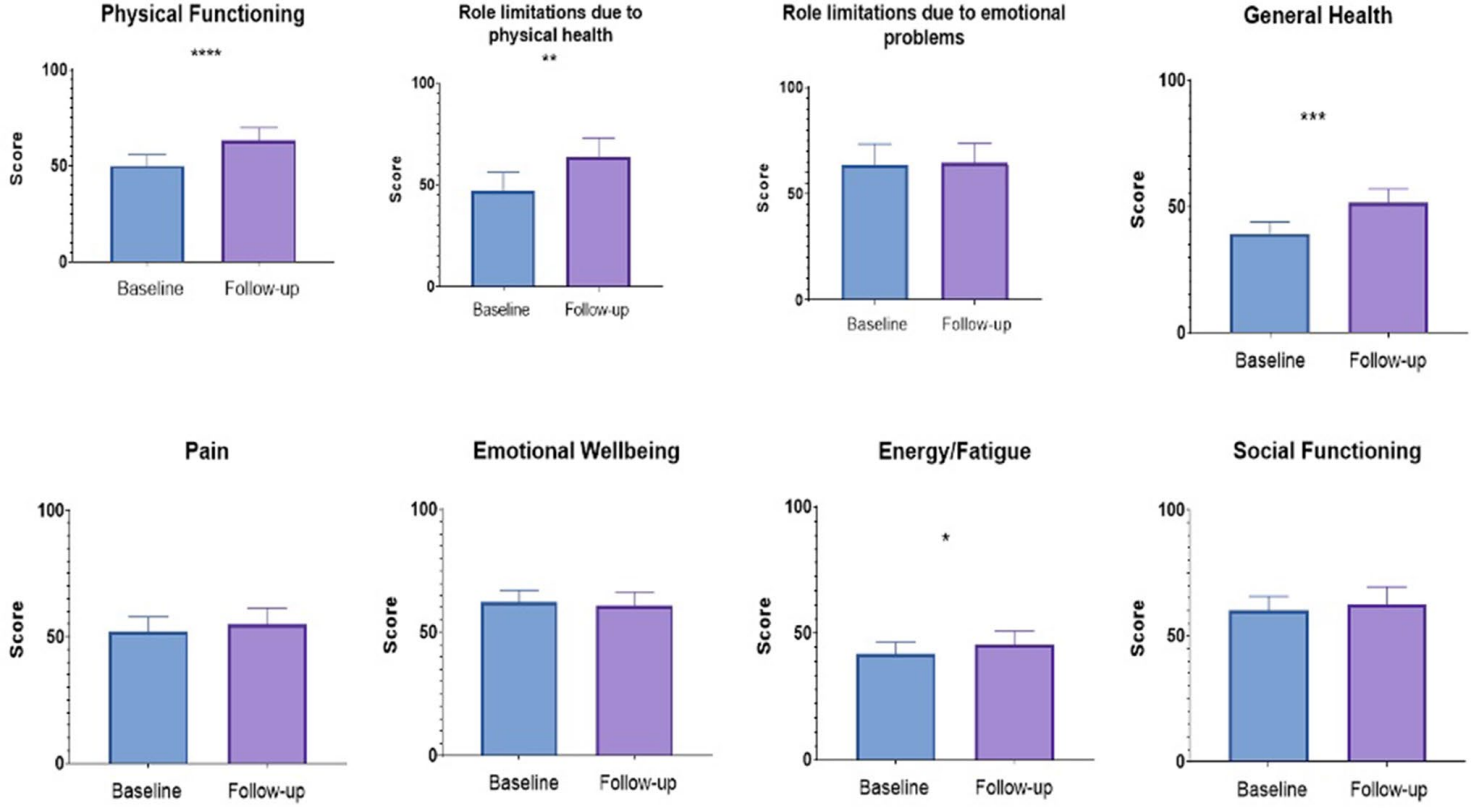
Baseline vs follow-up mean RAND SF-36 domain scores. ****P < 0.001, ***P < 0.005, **P < 0.01, *P < 0.05

Scoring of SF-36 questionnaire responses demonstrated significant improvements in postoperative domain mean scores for physical functioning and role limitations due to physical health and general health (Fig. 2). The remaining domain scores for role limitation due to emotional problems, pain, fatigue, emotional wellbeing, and social functioning did not demonstrate statistically significant differences at postoperative follow-up compared to baseline.

### Beck Depression Inventory

**Fig. 3:**
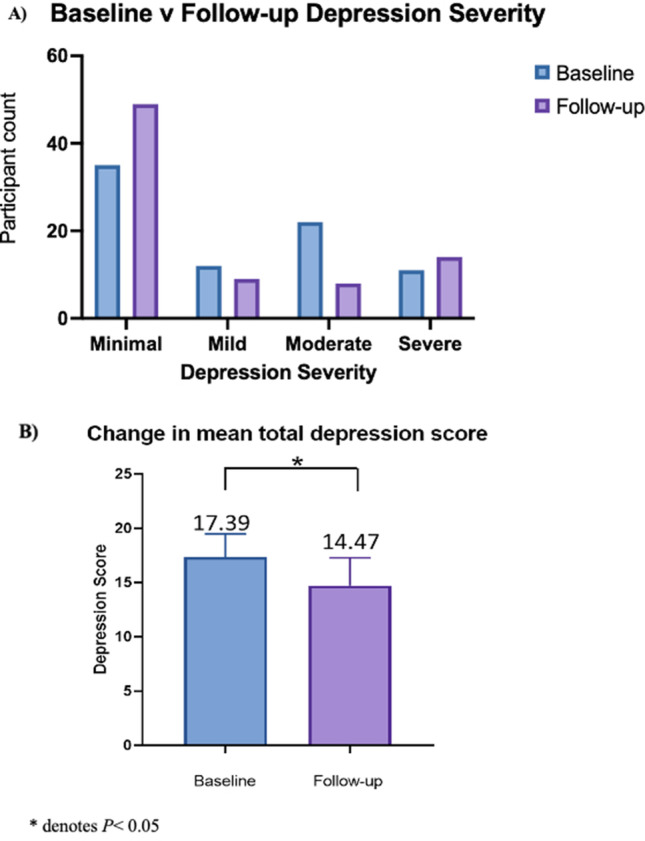
Change in depression severity index scores and severity

Fifty-six percent of respondents self-reported a diagnosis of depression at baseline compared to 51% at time of follow-up (Table 2). Depression scores were reduced from a mean of 17.35 (9.57) at baseline to 14.7 (11.57), *P* = 0.037 (Fig. 3).

### Dysphagia

**Fig. 4:**
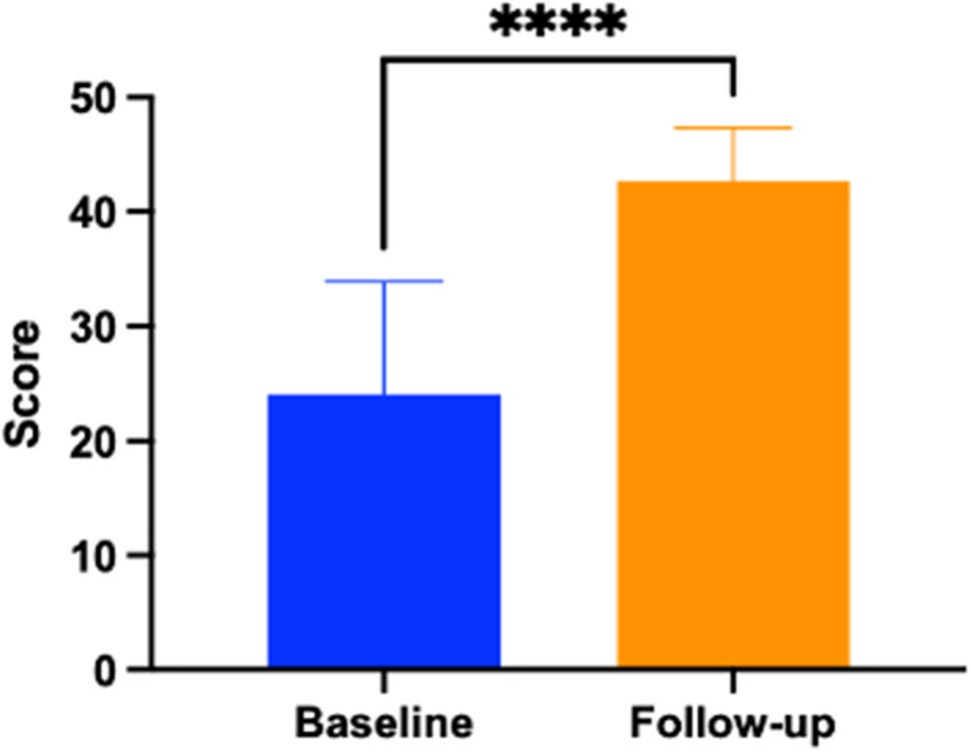
Change in mean Dakkak dysphagia severity (****P < 0.001)

Dakkak scores demonstrated a statistically significant mean increase in Dakkak dysphagia score post bariatric surgery, of 18.5units (10.39); *P* < 0.0001 (Fig. 4) with 95.1% of our sample experiencing dysphagia on follow up compared with 27% at baseline.

### Patient Satisfaction

Just over half of participants (53.9%) were satisfied with their bariatric procedure, 37.1% were dissatisfied, and 9% were neither satisfied nor dissatisfied. Participant satisfaction with surgery correlated significantly with TWL and change in depression score at follow-up (adjusted *R*^2^ 23.1%). Each kilo weight loss was associated with an improved satisfaction score of 0.055 units (CI_95%_ 0.025–0.084), *P* < 0.001, while each unit increase in depression score at follow-up demonstrated a 0.095 unit decrease in patient satisfaction (CI_95%_ − 0.159 to − 0.031; *P* = 0.004). There was no correlation between participant satisfaction and follow-up Dakkak dysphagia scores (Pearson = 0.03 (CI_95%_ − 0.050–0.107; *P* = 0.472)).

Two thirds of participants (65.9%) responded that they would choose to have the bariatric surgery procedure again, 22.7% responded that they would not have the bariatric surgery procedure again, and 11.4% of participants were unsure if they would make the same choice again.

### Relationship Between Adjusted Quality of Life and Covariates

Bivariate correlation analysis of follow-up AQoL utility scores with pre- and postoperative change in weight and patient satisfaction is summarized in Table 3. There was a statistically significant positive relationship between follow-up AQoL utility scores and TWL, and between follow-up AQoL utility scores and patient satisfaction. Each kilo weight loss translated to an improved AQoL utility score of 0.251 units (CI_95%_ 0.025–0.452; *P* = 0.03). Patient satisfaction demonstrated an improved mean AQoL utility score of 0.581 units (CI_95%_ 0.408–0.714; *P* < 0.001). Conversely for each increased unit of BDI-II depression score, a 0.40 unit drop was seen in post operative AQoL-utility score (CI_95%_ − 0.577 to − 0.187; *P* < 0.001). There was no correlation between increase in Dakkak dysphagia score and follow up AQoL utility score, *P* = 0.59.

**Table 3:** Univariate correlation analysis of follow up AQoL utility and covariates

	Pearson	*P*	95% CI
Change in BDI-II score	− 0.40	< 0.001	− 0.577 to − 0.187
Change in Dakkak score	− 0.063	0.599	-
Satisfaction with surgery	0.581	< 0.001	0.408 – 0.714
% Total weight loss	0.251	0.03	0.025 – 0.452

These correlations were further evaluated with multiple stepwise linear regression analysis (Table 4) which showed a significant relationship between change in depression score and patient satisfaction score (adjusted R^2^ of 37%). As depression score increased AQoL utility score decreased by 0.005 units (CI_95%_ − 0.009 to − 0.001; *P* = 0.029). A significant positive relationship was shown between patient satisfaction and follow-up AQoL utility score. As patient satisfaction increased, AQoL utility score increased by 0.036 units (CI_95%_ 0.022–0.05; *P* < 0.001).

**Table 4:** Multiple linear regression analysis follow-up AQoL utility and covariates

	Beta	*P*	95% CI
Change in BDI-II score	− 0.005	0.029	− 0.009 to − 0.001
Change in Dakkak score	0.094	0.319	-
Satisfaction with surgery	0.036	< 0.001	0.022–0.051
% Total weight loss	0.07	0.492	-

## Discussion

Our results show that sustained weight loss following bariatric surgery at a mean of 5.7 years follow-up is associated with improved HRQoL scores across the SF-36, AQoL-8D, and Beck Depression Inventory. Improvements in HRQoL utility scores correlated with patient satisfaction and depression scores. Most patients experienced an increase in dysphagia (from none to mild); however, this did not appear to impact patient satisfaction. On average, participants gained 3.2 (1.67) QALYs between baseline and follow-up.

These positive changes were achieved with a mean 18.5 kg weight loss (13.57% TBWL) at follow-up. This is a more modest weight loss than we have previously described at 5 years [29] but exceeds the level of weight loss we have previously shown to be correlated with significant health benefits at a threshold of 10%TBWL [27, 30].

The flow on health economic effects of improved HRQoL in patients who undergo an obesity health intervention which results in clinical improvement in previously diagnosed depression has been shown to yield an 8.8% reduced risk of being a high health care user [8]. Our findings are similar to the published literature as post bariatric surgery there was a statistically significant improvement in depression severity despite only modest maintained weight loss [32]. Our data also demonstrated that patient satisfaction with their bariatric surgery is a significant indicator of postoperative HRQoL. Increased dysphagia did not correlate significantly with MAU scores, which is perhaps demonstrative of the significant QoL gains to be made following bariatric surgery that may overshadow less desirable side effects of the procedure [33]. Patient satisfaction responses further support this, correlating significantly with TWL and depression in keeping with previously published data [27]. Just over half the participants were satisfied with the bariatric procedure and two thirds would in hindsight repeat the same surgery.

Among the HRQoL assessments, the follow-up AQoL-8D questionnaire demonstrated significant improvement in both the mental and physical super dimension scores. In contrast, SF-36 physical QoL scores improved whereas SF-36 mental QoL scores did not demonstrate significant change. This is consistent with other studies at time points > 5 years [27, 31]. AQoL-8D has greater coverage of mental and social dimensions of health, compared to the SF-36 tool [27, 31]. The AQoL-8D, therefore, offers significant advantages for evaluation of psychosocial dimensions of health and works synergistically with the SF-36 questionnaire to demonstrate QoL trends with a higher correlation with subjective wellbeing [13].

The QALY is an important metric that facilitates understanding of the total potential benefits of a therapeutic intervention by combining the HRQoL benefits with how much the intervention would extend a patient’s life. Its unit of measurement is reliably translatable to the cost utility of therapies for individuals with different diseases [34]. As such, QALYs inform government and health provision bodies on how to allocate a limited budget to different therapies and health services [9, 35]. Participants demonstrated a statistically significant mean gain of 3.2 (1.67) QALY at the postoperative follow-up. However, it is beyond the scope of this study to attribute cost utility due to limited sample size.

Our study has several limitations. First, this was a single institution analysis of a small sample size from one country in a government-funded hospital setting, and as such, results may not translate to other populations. However, this is also the first prospective study to perform long-term follow-up in this patient demographic of bariatric surgery. Consistent with most follow-up survey studies, there was significant loss to follow-up, with 45% of consenting participants returning completed questionnaires. Reported loss to follow-up in QoL/psychological studies in individuals with obesity is approximately 30% with female gender a risk factor for increased attrition [36, 37]. Participant attrition in this study was 24/180 (13%) at the 4-week phone call by investigators to return questionnaires and occurred prior to the COVID-19 pandemic declaration. Following the second follow-up phone call, seventy-five patients failed to return the questionnaires. The second reminder (at 8 weeks) and subsequent deadline (at 16 weeks) for participants to return the follow-up questionnaires fell within the declaration of the COVID-19 pandemic and associated government restrictions being placed in Australia. As such, participants may have placed study participation as a lower priority and significantly impacted participant attrition.

In addition to limiting study power, this was another source of potential bias. The measurement of HRQoL outcomes was based on patient reported assessment responses, and there is a possibility of response bias. A further limitation is that we were unable to perform comparisons between different types of bariatric surgery, as LAGB insertion was the predominant procedure at our institution between 2013 and 2016. However, the adjustable gastric band offered HRQoL improvement despite more modest weight loss than might be expected from other procedures at a mean duration of 5.7 years of follow-up, similar to the physical health benefits we have previously demonstrated [38]. Translatability at a system level, relating broadly to the application of bariatric surgery as a therapy rather than a single procedure represents a strength of this study. Due to the duration of the study and location within the more constrained government-funded healthcare system, it was not possible to perform such an evaluation across different procedures.

Our pragmatic study using prospectively collected data provides real-world insight into the quality of life outcomes that can be achieved following bariatric surgery in Australia’s public hospital system. This is the first prospective study to used validated MAU (AQoL-8D), MA (SF-36), and disease-specific (BDI-II, Dakkak) self-assessment instruments to compare baseline and longer term quality of life following bariatric surgery. Future larger, multi-center prospective studies that include resectional bariatric surgery are needed to further explore HRQoL and QALY changes following bariatric surgery, in a variety of healthcare settings, with translation of the intervention’s cost utility.

## Conclusion

Our single-center study found that sustained weight loss at a mean of 5.7 years following bariatric surgery was associated with substantial and significant improvement in physical and mental health and significant QALY gains. The improved HRQoL following bariatric surgery correlated significantly with patient depression and satisfaction with surgery and resulted in a significant improvement in QALYs. These findings can help inform future research to ensure healthcare systems deliver high-value obesity interventions that improve patient-centered outcomes.
